# Children with Low Handgrip Strength: A Narrative Review of Possible Exercise Strategies to Improve Its Development

**DOI:** 10.3390/children9111616

**Published:** 2022-10-25

**Authors:** Takashi Abe, Robert S. Thiebaud, Hayao Ozaki, Sakiya Yamasaki, Jeremy P. Loenneke

**Affiliations:** 1 Graduate School of Health and Sports Science, Institute of Health and Sports Science and Medicine, Juntendo University, Inzai-shi 270-1695, Chiba, Japan; 2 Division of Children’s Health and Exercise Research, Institute of Trainology, Fukuoka-shi 814-0001, Fukuoka, Japan; 3 Department of Human Performance and Recreation, Brigham Young University–Idaho, Rexburg, ID 83440, USA; 4 School of Sport and Health Science, Tokai Gakuen University, Miyoshi-shi 470-0207, Aichi, Japan; 5 Department of Human Sciences, Seinan Gakuin University, Fukuoka-shi 814-8511, Fukuoka, Japan; 6 Department of Health, Exercise Science, and Recreation Management, Kevser Ermin Applied Physiology Laboratory, The University of Mississippi, Oxford, MS 38677, USA

**Keywords:** aging, development, grip strength, muscular strength, human baseline

## Abstract

Background: Handgrip strength (HGS) is a predictor of health in both children and adults. Evidence suggests that without a possible strategy, children with low HGS may become adults with low HGS. However, little is known about what strategies are effective for children with low HGS to achieve a higher baseline level in adulthood. This narrative review aimed to investigate whether physical exercise interventions could improve HGS in children. Methods: The relevant databases/search engine was searched using keywords related to the main topics discussed throughout this review. Results: Our findings suggest that it may not be possible to improve HGS over that observed from normal development with physical education or traditional resistance-training programs. However, if the training program includes exercises that directly stimulate the forearm/hand muscle groups to grip, it may be possible to obtain changes in HGS that exceed the changes due to normal developmental growth. Conclusion: Although there are associations between HGS and markers of health, no research could be identified that examined whether increasing HGS would lead to an improvement in health. If an increase in HGS really does represent an improvement in long-term health, then gripping exercise may need to be included into physical activity programs during the growth/development phase.

## 1. Introduction

Muscular strength is a powerful predictor and biomarker of current and future health, regardless of age or sex. For example, low handgrip strength is a predictor of future disability [1,2] and mortality [3,4] in middle-aged and older adults. In children and adolescents, low handgrip strength is also associated with poor cardiometabolic and bone health outcomes [5,6] and premature death [7]. Therefore, it may be useful to acquire and maintain a high level of muscular strength throughout life [8].

Our recent study using a Smedley dynamometer suggests that handgrip strength can be measured accurately in children as young as four [9,10]. At this age, individual differences in muscle strength are already observed. Several studies reported that children with low muscular strength are likely to become young adults with low muscular strength [11,12]. For example, a twenty-year follow-up study showed that children with low handgrip strength were approximately five times more likely to have low handgrip strength as young adults compared with children who had high handgrip strength [13]. This evidence suggests that without a possible strategy, children with low muscular strength are likely to become adults with low muscular strength.

Although fetal environment [14,15], protein intake [16], daily physical activity [17], and structured resistance exercise [18,19] are major factors affecting a child’s low muscular strength, there are only a limited number of strategies that children can actually take after birth. Unfortunately, little is known about which strategies are effective for children with low muscular strength to achieve a higher baseline level of muscular strength [8], especially handgrip strength, by adulthood. For adults, exercise training (i.e., resistance-type exercise) is recommended for developing and/or maintaining muscular strength [20]. Resistance exercise for children and adolescents includes lifting weights as well as various bodyweight movements and upper-body exercises that take place in play from an early age [21]. These physical activities are expected to contribute to the development of upper-body and lower-body muscular strength including handgrip strength for children and adolescents. Thus, the aim of this narrative review was to investigate the potential of exercise training as a way for children with low muscular strength to improve their strength. We also examined whether the effects of exercise training are affected by the age at which they are performed.

## 2. Materials and Methods

A literature search was performed using the electronic databases (PubMed and Scopus) and a search engine (Google Scholar) through September 2022. The search consisted of keywords related to the main topics discussed throughout this review: physical activity or resistance training and muscular strength and/or body composition. An example search strategy for PubMed, Scopus, and Google Scholar is provided (Appendix A). References from pertinent articles and the names of the authors cited were cross-referenced to locate any further relevant articles not found with the initial search. In previous studies, jumping ability, such as the standing long jump, has been used as an index of muscular strength in children. As mentioned above, however, handgrip strength has been used as a predictor of current and future health. Therefore, this review focused specifically on changes in handgrip strength following interventions.

In children and adolescents, physical activity is a key component to promote normal growth and development [22]. Quantitative and qualitative changes in muscle are constantly observed during this period. Therefore, to assess the effects of exercise on developing children and adolescents, it is necessary to compare changes in the intervention and control groups. In this narrative review, we excluded cross-sectional studies and included only intervention studies with a control group. In addition, the study needed to include individuals under the age of 18 and to report changes in the absolute value of handgrip strength.

## 3. Findings

### 3.1. Effect of Family- and School-Based Physical Activity Interventions on Handgrip Strength

Family-based and school-based interventions are both strategies for increasing physical activity in children and adolescents. Preschool children spend considerable time under parents’ care, thus, family-based physical activity promotion programs are an important strategy to improve strength. Similarly, physical activity promotion is predominantly conducted in schools. The effects of school-based interventions are being investigated by comparing normal curricula with extra physical education lessons.

Regarding family-based programs, Labayen et al. [23] compared differences in changes in physical fitness (e.g., handgrip strength, 20 m shuttle run, standing long jump), body composition (as measured by dual-energy x-ray absorptiometry (DXA)), and cardiometabolic and diabetes risk factors between two groups (mean age 10.6 ± 1.1 years of age): one group (*n* = 57) received a family-based lifestyle educational program (control), and another group (*n* = 49) received the same intervention plus an exercise program (90 min session involving aerobic and resistance exercise, three times per week). Following 22 weeks of the intervention, statistically significant increases were observed in both groups for all physical fitness variables, but no statistically significant differences were observed between groups in markers of physical fitness (i.e., change was not greater with intervention). For example, the pre-post change in handgrip strength calculated using reported values was 2.4 kg in the intervention with exercise group and 1.8 kg in the control group. Although family-based interventions do result in consistent increases in moderate and vigorous physical activity in the children [24], this improvement may not lead to increases in muscular fitness beyond that observed with normal development.

Several studies have investigated the effects of school-based physical activity interventions on body composition, physical fitness, and cardiovascular risk factors. For example, Rexen et al. [25] examined the effect of extra physical education lessons on children’s development of physical fitness (e.g., handgrip strength, shuttle run, vertical jump). All primary schools in one area were invited to participate in the project as schools with extra physical education, but only six attended extra physical education lessons (normal curriculum plus 270 min/week divided over at least 3 sessions per week) and the rest maintained their normal curriculum (90 min per week). Participants consisted of children across 5 age groups from preschool to fourth grade (mean age 8.2 years), with a follow-up of 2.5 years. The authors reported a greater improvement in the composite fitness score of the older children (third and fourth grades) who performed extra physical education lessons compared to the corresponding children in normal physical education lessons. However, children within the younger grades (preschool, first, and second grades) had no effect of extra physical education lessons on the development of composite fitness scores over time. Since they only reported total fitness scores, the impact of extra physical education lessons on handgrip strength development is unknown, but no change in total score suggests no change in handgrip strength.

Bogataj et al. [26] also compared the changes in physical fitness (handgrip strength, countermovement jump, medicine ball throw, and aerobic performance test) and body composition (as estimated by bioelectrical impedance) between the school-based exercise/nutrition intervention group (*n* = 24, mean age 15.5 years) and a control group (*n* = 24, mean age 15.7 years). The exercise/nutrition group performed circuit training three times a week for eight weeks, and each session consisted of ten different exercises using their own body weight. Each exercise was performed for 30 s and then followed by a 15-s rest period. Two circuits were completed for a total time of 15 min. The children and their parents also participated in the nutrition intervention program. The control group was instructed to maintain physical education activities and not change their diet. After eight weeks, there were significant improvements in all physical fitness variables. The only difference between groups was that the exercise/nutrition group improved more in the medicine ball throw and the aerobic performance test. Of note, there were no statistically significant differences in handgrip strength (pre-post change: intervention group = 0.54 kg, control group = 0.52 kg; Time effect: *p* = 0.004; Interaction: *p* = 0.949). Other studies have also reported statistically significant improvements in physical fitness variables in a school-based intervention group compared with a control group [27,28,29,30], but there was no effect of extra physical education programs on handgrip strength, although research was limited [25,26,31].

### 3.2. Effect of Upper Body Resistance Training on Handgrip Strength

The American Academy of Pediatrics reported that if training programs are well supervised with an emphasis on proper technique, then children and adolescents can gain muscular strength with resistance training with low injury rates [21]. The increases in strength are primarily attributed to neurological mechanisms (e.g., enhanced motor unit recruitment) [21]. As mentioned above, therefore, resistance training in children and adolescents is expected to contribute to the development of handgrip strength following upper-body resistance training (including grip training). For example, Faigenbaum et al. [32] compared the effects of different training frequencies (once a week, twice a week, and an age-matched control without training) of whole-body resistance exercise on upper- and lower-body muscular strength in boys and girls between the ages of 7 and 12 years old. Both training groups performed a single set of 10–15 repetitions on 7 upper body (seated chest press, chest crossover, lat pull down, seated low, shoulder press, biceps curl, and triceps extension), 3 lower-body (leg press, leg extension, and leg curl), and 2 trunk (abdominal curl and lower back extension) exercises using child-size strength training equipment for 8 weeks. Compared with the non-exercise control group (*n* = 13), dynamic (1 repetition maximal (1RM)) leg press strength increased significantly in both once a week (*n* = 22) and twice a week (*n* = 20) training groups. Chest press strength (1RM) was observed to increase significantly only in the twice-weekly group compared to the control. However, there were no statistically significant differences (*p* > 0.05) in handgrip strength (pre-post change: once a week = −0.1 kg, twice a week = 2.4 kg, control = −0.7 kg) and jump performance among the groups following the training program. This result is consistent with that observed in adults, where handgrip strength may not change significantly following traditional resistance training for the major muscle groups throughout the body [33,34].

Hand grip exercise training, which has a direct effect on the forearm/hand muscles, is expected to be involved in improving handgrip strength [35]. For example, Siegel et al. [36] investigated the effects of resistance training on handgrip strength in an experimental group by comparing it with a control group. The experimental group (26 boys and 24 girls, mean age 8.4 years) performed upper body exercise using hand-held weights, stretch tubing, balls, and self-supported movements (e.g., wheelbarrow, sealwalk, crabwalk) three times a week. The control group (30 boys and 16 girls, mean age 8.6 years) had a free-play period which was part of the normal school routine. Following a 12-week intervention, the changes in handgrip strength were significantly greater (*p* < 0.05) for the experimental group compared to the control group (pre-post change in right hand: experimental group = 1.5 kg and control group = 0.3 kg). Karatrantou et al. [37] also examined the effects of hand squeezing exercise training using hand therapy balls in young wrestlers twice a week for four months. The hand training group (18 children and 18 adolescents) performed about three quarters of an hour of wrestling training and then about a quarter of an hour of hand squeezing training, while the control group (18 children and 18 adolescents) performed wrestling training for about one hour. The authors reported that maximal handgrip strength increased significantly (*p* < 0.001) after two and four months compared to the pre-training value in both children (preferred hand—Training group: pre = 16.3 kg, 2 months = 18.3 kg, and 4 months = 24.3 kg; Control group: pre = 15.2 kg, 2 months = 16.7 kg, and 4 months = 21.1 kg) and adolescents (preferred hand—Training group: pre = 37.8 kg; 2 months = 40.4 kg; 4 months = 46.8 kg; Control group: pre = 37.6 kg, 2 months = 39.1 kg, and 4 months = 40.4 kg) in both groups. After four months of training, however, the handgrip strength of the hand training group was higher (*p* < 0.001) than that of the control group in both children and adolescents. Recently, one study reported the effects of a six-month upper-body and lower-body exercise training intervention on handgrip strength in preschool-aged children [38]. Although the specific exercise related to improving handgrip strength was not described, significant improvement (*p* < 0.01) in handgrip strength was confirmed in the intervention group (pre-post change; 1.7 kg) compared to the control group (pre-post change; 1.1 kg). In contrast, after a 10-week lower-body training intervention, which included mainly jump-type exercises, no significant difference (*p* = 0.458) was observed in the change in handgrip strength between the control (pre-post change; 1.0 kg) and intervention (pre-post change; 0.7 kg) groups [39]. Similarly, despite a significant change in aerobic capacity after a 12-week concurrent training (moderate-intensity treadmill running training combined with resistance training (MICT + RT) or high-intensity treadmill interval training combined with resistance training (HIIT + RT)) intervention, handgrip strength did not change in the intervention (pre-post change; MICT + RT = −1.1 kg and HIIT + RT = 0.9 kg) and control (pre-post change: −1.3 kg) groups [40]. Previous studies reported increased muscle strength of the untrained upper extremities due to resistance training of the lower extremities (called the cross-education or cross-transfer effect) [41,42]. However, there are no studies of increased handgrip strength with the above lower-body training intervention.

## 4. Conclusions and Future Tasks

The majority of studies (though not all [38]) suggest that it may not be possible to improve handgrip strength over that observed from normal development with physical education or traditional resistance training programs. Unfortunately, most studies investigating school-based physical education effects did not include handgrip strength changes after exercise interventions. However, if the training program includes exercises that directly stimulate the forearm/hand muscle groups to grip, it may be possible to obtain changes in handgrip strength that exceed the changes due to normal developmental growth. One thing to consider is that the training period was within half a year for a majority of studies, and the descriptions of the exercise programs were not always entirely clear.

Although there are associations between handgrip strength and markers of health, it is still unknown if increasing handgrip strength would lead to an improvement in health. It is possible that handgrip strength, only in the absence of direct training, represents an individual’s future health risk. For example, while gripping exercise does improve handgrip strength, traditional resistance exercise does not have a measurable influence. Given that a variety of interventions fail to improve handgrip strength, this might suggest that children may not be able to measurably alter their long-term strength potential. In other words, a change in strength could be observed if trained directly, but this might not have any influence on an individual’s health status. On the other hand, if an increase in handgrip strength really does represent an improvement in long-term health (as the associations might suggest), then gripping exercise may need to be included in physical activity programs during the growth and development phase [7]. The mechanism explaining the inverse association between handgrip strength and morbidity/mortality remains unclear. However, genetic [43] and non-genetic [3,4,44,45] factors have been proposed to explain these associations. Currently, it is unknown how changing these biomarkers during the growth period can impact future morbidity and mortality. Additional longitudinal studies are needed to better address these questions.

## Data Availability

Not applicable.

## Appendix A

Sample search strategy using databases/search engine.
